# Dietary Flavonoids Luteolin and Quercetin Inhibit Migration and Invasion of Squamous Carcinoma through Reduction of Src/Stat3/S100A7 Signaling

**DOI:** 10.3390/antiox8110557

**Published:** 2019-11-15

**Authors:** Jhen-Jia Fan, Wen-Hsien Hsu, Kuen-Haur Lee, Ku-Chung Chen, Cheng-Wei Lin, Yu-Lin A Lee, Tzu-Ping Ko, Lang-Ta Lee, Ming-Ting Lee, Mau-Sun Chang, Chia-Hsiung Cheng

**Affiliations:** 1 Institute of Biochemical Sciences, National Taiwan University, Taipei 10617, Taiwan; jhenjia@gmail.com; 2 Food and Drug Administration, Ministry of Health and Welfare, Taipei 11561, Taiwan; 3 Department of Surgery, Wan-Fang Hospital, Taipei Medical University, Taipei 11034, Taiwan; angiohsu@gmail.com; 4 Graduate Institute of Cancer Biology and Drug Discovery, College of Medical Science and Technology, Taipei Medical University, Taipei 11034, Taiwan; khlee@tmu.edu.tw; 5 Cancer Center, Taipei Medical University Hospital, Taipei Medical University, Taipei 11034, Taiwan; 6 Department of Biochemistry and Molecular Cell Biology, School of Medicine, College of Medicine, Taipei Medical University, Taipei 11034, Taiwan; kuchung@tmu.edu.tw (K.-C.C.); cwlin@tmu.edu.tw (C.-W.L.); 7 Departments of Medicine and Pediatrics, Duke University Hospital, Durham, NC 27704, USA; amy.lee@duke.edu; 8 Institute of Biological Chemistry, Academia Sinica, Taipei 11529, Taiwan; kotping@gate.sinica.edu.tw (T.-P.K.); mtlee@gate.sinica.edu.tw (M.-T.L.); 9 Department of Nursing, Ching Kuo Institute of Management and Health, Keelung 20301, Taiwan; garfi@ems.cku.edu.tw

**Keywords:** luteolin, quercetin, S100A7, Src, Stat3, metastasis

## Abstract

Flavonoids are well-known antioxidants and have shown the ability to prevent tumor formation and recurrence. Especially in dietary flavonoids, they have provided convenience and consistence of intake for long-term prevention of tumor formation. Previous reports suggested that S100 calcium-binding protein A7 (S100A7) might activate epithelial–mesenchymal transition (EMT) signaling and promote the metastasis of tumor cells; however, the regulatory signaling was unclear. In this study, we found that S100A7 was highly expressed in cancer cells and could be reduced by luteolin (Lu) and quercetin (Qu) through Src/Stat3 signaling. We found that the protein levels of S100A7, phosphorylated Src (p-Src), and p-Stat3 were increased in A431-III cells. Flavonoids Lu and Qu reduce protein levels of p-Src, p-Stat3 and S100A7 in A431-III cells. Treatment of A431-III cells with Src inhibitor SU6656 and Stat3 inhibitor S3I-201 also reduced the protein levels of S100A7. Transactivation activity of 5′-upstream regions of *S100A7* was activated by Stat3 but was reduced by treatment with Lu, Qu, SU6656 and S3I-201. The treatment also reduced the migratory and invasive abilities of A431-III cells. In a further analysis of EMT markers, the protein level of E-cad increased and that of Twist decreased after treatment with the inhibitors and flavonoids. Overexpression of S100A7 decreased the protein level of E-cad and increased the Twist level, whereas knockdown of S100A7 had the opposite effects. Treatment with S3I-201, Lu and Qu, compared to the control, were found to decrease metastasis of tumor cells in zebrafish larvae. These results suggest that Lu and Qu may inhibit Src/Stat3/S100A7 signaling to reduce tumorigenesis of cancer cells.

## 1. Introduction

The flavonoids are components in fruits and plants and show anti-oxidant, anti-inflammatory and anticancer activities [1,2,3,4,5,6,7,8,9,10,11,12]. The antioxidant activity of flavonoids is due to their ability to decrease free radical formation and to scavenge free radicals. It is worth noting that the structure-activity was established in the relationships with antioxidant activity [13]. In addition, we have also established the structure-activity relationships between luteolin (Lu), quercetin (Qu) and protein kinase [14]. Reports suggested that Lu and Qu are the most potent plant flavonoids in terms of their biological activities [14,15]. The antioxidant activity of Lu and Qu regulated reactive oxygen species (ROS) signaling and induce apoptosis in human cancer cells [16,17,18,19,20,21]. The metastatic inhibition of flavonoids on cancer cells have also been discussed [22,23,24,25]. In our earlier reports, we have established a highly invasive A431-III cell line from parental A431 cell line to investigate the migrative and invasive mechanism in cancer cells [26]. Lu and Qu are shown to efficiently increase catalase expression and reduction of H_2_O_2_ production to decrease ROS stress in cancer cells [2]. Inhibition metastasis of A431-III cells by Lu and Qu were also observed. Further investigation of the inhibition mechanism of migration and invasion found that Lu and Qu inhibited Src/FAK/p-cortactin, Akt/mammalian target of rapamycin (mTOR) activated RPS12 and RPS19 signaling and UBE2S activated epithelial–mesenchymal transition (EMT) signaling [27,28,29,30]. Stat3 is a transcription factor activated by kinases to regulate the transcription of downstream genes. Reports suggested that JAK2/Stat3 signaling directly activates transactivation activity of the S100A7 promoter in normal human keratinocytes [31]. Stat3 phosphorylation activated Oncostatin M and the downstream S100A7 gene expression in breast cancer [32]. Blockading of Stat3 inhibited cytokines-induced S100A7 expression in breast cancer cells [33].

As a member of the S100 gene family, S100A7 (S100 calcium-binding protein A7, psoriasin) promoted the metastatic abilities of cancer cells [34,35]. S100A7 induced different effects in association with the various progression stages of tumors. S100A7 was shown to regulate tumorigenesis in cancer cells. In pre-malignancy carcinoma, S100A7 is highly expressed to increase β-catenin degradation. S100A7 overexpression is also associated with the differentiation of the glandular epithelium of pre-invasive ductal carcinoma in situ (DCIS) [36]. S100A7 is one of the most highly expressed genes in cancer tissues compared to normal breast tissue, where its expression is almost undetectable according to the serial analysis of gene expression (SAGE) database of the Cancer Genome Anatomy Project [37]. Up-regulation of S100A7 by stable S100A7 transfection increases nuclear factor (NF)-κB binding activity as well as matrix metalloproteinase (MMP)-9 and vascular endothelial growth factor (VEGF) expression, whereas the down-regulation of S100A7 by small interfering (si)RNA in S100A7 complementary (c)DNA-transfected MDA-MB-468 cells decreases NF-κB binding activity and MMP-9 and VEGF expression. The S100A7 gene controls the invasive potential of human MDA-MB-468 cells and their proliferation through the regulation of the NF-κB activity and its target genes [38]. S100A7 is differentially up-regulated in three of six cervical cancer patients [39]. S100A7 is reported to promote migration, invasion and metastasis of human cervical cancer cells through epithelial-mesenchymal transition (EMT) [40] but the activation signaling of S100A7 is unknown. 

Previously, we also showed that the metastasis inhibition of A431-III cells by the flavonoids, Lu and Qu through the reduction of Src phosphorylation [41]. In this study, we identified the function of Lu and Qu on inhibition Src/Stat3/S100A7 signaling in squamous carcinoma cells. The analysis of protein levels of S100A7 expressed in A431-III and A431-P by treatment with Lu and Qu. Src and Stat3 inhibitors were used to analyze the activation signaling of S100A7. A luciferase assay was used to analyze the transactivation inhibition of S100A7 promoter by Lu and Qu. A wound-healing assay was used to investigate the effects of S100A7 on the migration of cancer cells after treatment with Lu, Qu, Su6656, and S3I-201. The protein levels of EMT markers were used to analyze the effects on the same treatment. Knockdown of S100A7 in A431-III cells and overexpression of S100A7 in A431-P cells were used to analyze the effects of S100A7 on EMT signaling. Zebrafish larvae were used to analyze the metastasis of tumor cells in vivo.

## 2. Materials and Methods

### 2.1. Chemicals and Reagents

RPMI-1640 and fetal bovine serum (FBS) were obtained from ThermoFisher Scientific (Cleveland, OH, USA). Anti-S100A7, anti-Stat3 and anti-phosphorylated Stat3 (anti-p-Stat3) antibodies were obtained from GeneTex (Irvine, TX, USA). The anti-Src antibody was acquired from Cell Signaling Technology (Danvers, MA, USA). Anti-p-Src (Y418), anti-Twist and anti-E-cadherin (E-cad) antibodies were obtained from Abcam (Cambridge, UK). Anti-glyceraldehyde3-phosphate dehydrogenase (GAPDH) and anti-β-actin antibodies were purchased from Santa Cruz (Capitola, CA, USA). The polymerase chain reaction (PCR) forward and reverse primers were purchased from Purigo Biotech (Taipei, Taiwan). Luteolin (Lu) was purchased from Toronto Research Chemicals (North York, ON, Canada). Quercetin (Qu) was purchased from Nacalai Tesque (Kyoto, Japan). Su6656, S3I-201, dimethyl sulfoxide (DMSO), and 3-(4,5-dimethylthiazol-2-yl)-2,5-diphenyl tetrazolium bromide (MTT) were purchased from Sigma-Aldrich (Merck, Darmstadt, Germany).

### 2.2. Cell Culture

The human squamous carcinoma cell lines A431-P (A431 cell line, ATCC CRL-1555) and A431-III (generated from A431 cell line in our laboratory) cultured methods follow those in our earlier report [26]. Briefly, the A431-P and A431-III cells were cultured in RPMI-1640 medium containing 10% FBS (ThermoFisher Scientific) in a 5% CO_2_ atmosphere at 37 °C.

### 2.3. Cell Viability Assay

A431-III cells (10^5^ cells/well) were used to seed in 48-well plates and were incubated with 0.1% DMSO, 10, 20 and 40 μM of Lu and 10, 20 and 40 μM of Qu for 24h. The cultural medium was removed and fresh medium was added with 5 mg/mL of (3-(4,5-Dimethylthiazol-2-yl)-2,5- diphenyltetrazolium bromide (MTT) (Merck) to the culture at 37 °C for 4h. The medium was then removed and 200 μL DMSO was added to resolve precipitation. A Spark multimode microplate reader (TECAN, Männedorf, Switzerland) measured the absorbance at a wavelength of 570 nm.

### 2.4. Preparation of Cell Lysates

The phosphate-buffered saline (PBS) was used to wash and collect tumor cells. These cells were then lysed in gold-lysis buffer (20 mM Tris-HCl, at pH 7.9), 1 mM ethylene glycol tetraacetic acid (EGTA), 0.8% NaCl, 0.1 mM β-glycerylphosphate, 1 mM sodium pyrophosphate, 10 mM NaF, 1 mM Na_4_P_2_O_7_, 1 mM Na_3_VO_4_, 10% glycerol, 1% Triton X-100, 1 mM phenylmethylsulfonyl fluoride (PMSF), 10 μg ⁄mL aprotinin, and 10 μg ⁄mL leupeptin). Supernatants were collected by centrifugation at 14,000× *g* for 20 min at 4 °C. Protein concentrations were quantified using a Bio-Rad protein assay kit (Bio-Rad, Hercules, CA, USA). All protein samples were stored at −80 °C.

### 2.5. Western Blotting

Protein samples were mixed with sample buffer (250 mM Tris-HCl, at pH 6.8, 10% sodium dodecylsulfate (SDS), 30% Glycerol, 5% β-mercaptoethanol, and 0.02% bromophenol blue) and boiled for 5 min. Proteins were separated by sodium dodecylsulfate polyacrylamide gel electrophoresis (SDS-PAGE) and were transferred to a polyvinylidene difluoride (PVDF) membrane (Millipore, Billerica, MA, USA). The membrane was blocked with 5% bovine serum albumin (BSA) for 1 h at room temperature, which was followed by incubation with the primary antibody overnight at 4 °C. After washing with PBST (PBS and 0.25% Tween-20), the membrane was incubated with a secondary antibody conjugated with horseradish peroxide (Millipore) for 1 h. The membrane was washed with PBST and detected using an enhanced chemiluminescence (ECL) reagent kit (Millipore) followed by exposure to Amersham Imager 600 imagers (GE, Pittsburgh, PA, USA). ImageJ software (http://rsb.info.nih.gov/ij/index.html, NIH, Bethesda, MA, USA) was used to analyze the relative quantification of the ECL signals.

### 2.6. Cloning of Full-Length cDNA of S100A7

TRIZOL (Thermo Fisher Scientific) was used to extract total RNA from A431-III cells. A MEGAscript T7 Transcription Kit (Thermo Fisher Scientific, Cleveland, OH, USA) was used to synthesize full-length cDNA from the total RNA of A431-III cells following the manufacturer’s instructions. A KAPA HiFi PCR Kits (Kapa Biosystems, Woburn, MA, USA) was used to amplify the coding regions of *S100A7* from cDNA. The following primer pairs were used for the PCR: S100A7-F (5′-GCA GGA TGG CCC AAT GGA ATC AGC-3′); S100A7-R (5′-TTC GCT TCT CAG CTC CTC ACA TGG-3′); S100A7-HindIII-F (5′- CGA AGC TTA TGA GCA ACA CTC AAG-3′); and S100A7-EcoRI-R (5′-ATG AAT TCC TGG CTG CCC CCG GAA-3′). The PCR products were cloned into pGEM-T vector (Promega, Madison, WI, USA) for sequencing. The coding regions of *S100A7* in the pGEM-T plasmid were digested with restricted enzymes *HindIII* and *EcoRI* and inserted into pcDNA3-Flag vector to create the pcDNA3-S100A7-Flag plasmid.

### 2.7. Luciferase Assay

The saturated phenol (Thermo Fisher Scientific, Waltham, MA, USA) was used to extract the genomic DNA from A431-III cells using. The National Center for Biotechnology Information (NCBI) database was used to identify the 5′-upstream 1551-bp length of *S100A7* as a promoter. A KAPA HiFi PCR Kit (Kapa Biosystems, Woburn, MA, USA) was used to amplify DNA fragments from genomic DNA. The following primer pairs were used for the PCR: S100A7-pro-F (5′-TGC TGC CCT TCA CAG TCT CCA GTG TCT ATG-3′); S100A7-pro-R (5′-GGA AGC GTC ACG AGT AGA AGG ATG AGT GAG-3′); S100A7-pro-NheI-F (5′-AAT GCT AGC TGC TGC CCT TCA CAG TC-3′); and S100A7-pro-HindIII-R (5′-TAC AAG CTT GGA AGC GTC ACG AGT AG-3′). The amplified DNA fragment was then cloned into the pGEMT-Easy vector (Promega, Madison, WI, USA), followed by sequence verification. The *S100A7* promoter in the pGEM-T plasmid was digested with *NheI* and *HindIII* and then cloned into the pGL3-Basic vector to create the pGL3-S100A7-pro plasmid. The pGL3-Basic or pGL3-S100A7-pro plasmid was transfected into A431-III cells using the PolyJet transfection reagent (SignaGen Laboratories, Rockville, MD, USA) according to the manufacturer’s instructions. The culture medium was replaced with medium that did or did not contain inhibitors at 24 h post-transfection. Total cells were harvested at 48 h post-transfection. Luciferase activity was monitored with Luciferase Assay Reagent (Promega) and detected by a Spark multimode microplate reader (TECAN, Mannedorf, Switzerland).

### 2.8. Cell Migration Assay

A431-III cells (5 × 10^5^ cells/well) were plated in six-well culture plates in RPMI-1640 containing 10% FBS. After 24, cell monolayers were wounded by manually scratching them with a pipette tip and washing with PBS. The monolayers were then incubated with RMPI-1640 containing 10% FBS and/or different concentrations of chemicals at 37 °C for 24 h. A phase-contrast Zeiss Axio Vert.A1 inverted microscope (Zeiss, Jena, Germany) and a Leadview 2800AM-FL camera (Leadview, Taipei, Taiwan) were used to capture the size of the wound. The experiments were repeated in triplicate for each treatment group.

### 2.9. Cell Invasion Assay

Transwell inserts with polycarbonate filters (BD Biosciences, Franklin Lake, NJ, USA) were coated with extracellular matrix (ECM, BD Biosciences) for 4 h at 37 °C. A431-III cells were pretreated with 0.1% DMSO, 10 μM of Su6656, or 400 μM of S3I-201 for 24 h and treated with trypsin (Corning, Oneonta, NY, USA) to collect tumor cells. The A431-III cells (2 × 10^5^) were added into each insert and cultured at 37 °C for 24 h. The 4% paraformaldehyde (Sigma, ST. Louis, MO, USA) was used to fix the insert and stain with 0.1% crystal violet (Sigma, ST. Louis, MO, USA). 

### 2.10. Knockdown of S100A7 by Short Hairpin (sh) RNA

Two *S100A7* (shS100A7, clone ID: TRCN0000373395 and TRCN0000373396) and control (shGFP, clone ID: TrcN0000072178) shRNA plasmids came from the National RNAi Core Facility at the Institute of Molecular Biology (Academia Sinica, Taipei, Taiwan). PolyJet transfection reagent (SignaGen Laboratories) was used to transfect the shRNA plasmids into A431-III cells according to the manufacturer’s instructions.

### 2.11. Zebrafish Metastasis Model

Zebrafish (*Danio rerio*) embryos were obtained from the Zebrafish Core Laboratory of Taipei Medical University and maintained at 28 °C. All animal experiments followed the approval of Animal Care and Use Committee or Panel (IACUC/IACUP) (protocol #LAC-2019-0249). The methods were in accordance with the approved guidelines. The migration assay of cancer cells in zebrafish followed that in an earlier report [42]. Briefly, 2-day post-fertilization (dpf) zebrafish larvae were treated with MS222. Cancer cells were pre-treated with 0.1% DMSO, 400 μM S3I-201, 20 μM Lu and 40 μM Qu for 24h and were then used 0.25% trypsin (Corning) to collect tumor cells. Tumor cells were stained with 34 mM Nile red (Sigma-Aldrich, Merck, Germany) for 20 min. After washing with PBS 3 times, tumor cells were resuspended with PBS. IM300 Microinjector (Narishige, Tokyo, Japan) was used to microinject tumor cells into the yolk of zebrafish larvae at 30 psi. After microinjection, zebrafish larvae were incubated at 28 °C for 1 h and then transferred to a 32 °C incubator for further culture. A phase-contrast Olympus IX70 microscope (Olympus, Tokyo, Japan) and a SPOT camera (Sterling Heights, MI, USA) were used to measure migrative tumor cells at caudal vein rear yolk extension at 5-dpf zebrafish larvae. 

### 2.12. Statistical Analysis

Results from three independent experiments are expressed as the mean ± standard deviation (SD). Statistical significance between the two groups was determined by an unpaired Student’s *t*-test. For comparison of more than two groups, a one-way analysis of variation (AVOVA) followed by Tukey’s test was used. A probability of *p* < 0.05 is indicated by *; *p* < 0.01 is indicated by **; and *p* < 0.001 is indicated by ***.

## 3. Results

### 3.1. S100A7 is more Highly Expressed in Cervical Cancer Patients and A431-III Cells Accompanied by Activation of Src/Stat3 Signaling

**Figure 1:**
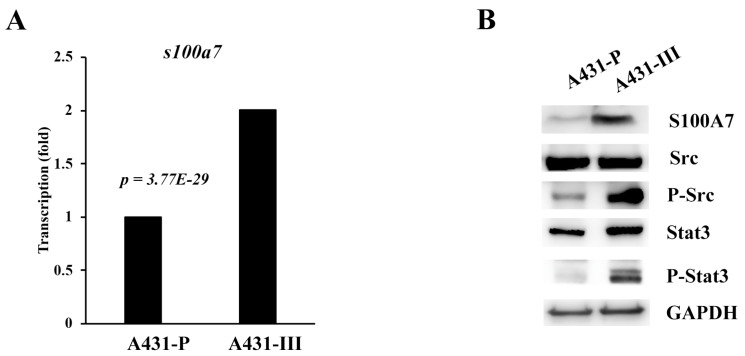
S100A7 is more-highly expressed in cervical cancer patients and A431-III cells accompanied by activation of Src/Stat3 signaling. (**A**) The mRNA levels of S100A7 in A431-P and A431-III cells, analyzed by microarray. (**B**) The protein levels of S100A7, Src, phosphorylated (p)-Src, Stat3 and p-Stat3 in A431-P and A431-III cells.

In our earlier reports, A431-III cells showed high migratory and invasive abilities [26,27,28,29,30,41]. From our microarray data [43,44], we found that the messenger (m)RNA levels of S100A7 were 2-fold higher in A431-III cells than in A431-P cells (Figure 1A). We also analyzed the promoter regions of S100A7 for its regulation. One Stat-binding site was found from the regions of -848 to -844. In our and others’ previous work, Src activated Stat3 and FAK/cortactin signaling and promoted migration and invasion of cancer cells [41]. In A431-III cells, the protein levels of S100A7, phosphor-Src and phosphor-Stat3 were higher than in A431-P cells (Figure 1B). These data suggest that high-level expression of S100A7 in cervical cancer patients and A431-III cells might be activated by Src/Stat3 signaling.

### 3.2. Luteolin (Lu) and Quercetin (Qu) Inhibit S100A7 in A431-III Cells by Suppressing Src/Stat3 Signaling

**Figure 2:**
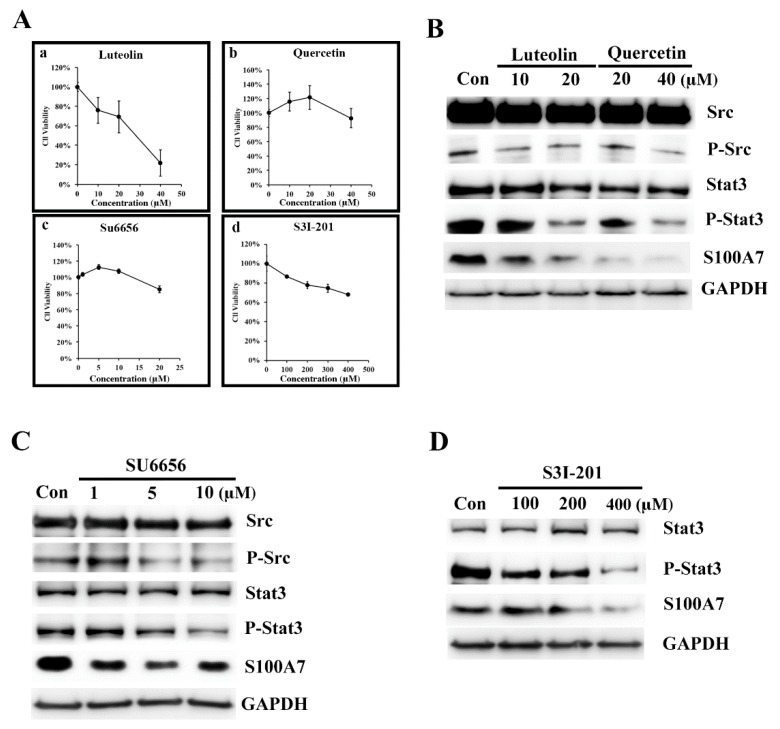
Luteolin (Lu) and quercetin (Qu) inhibit S100A7 in A431-III cells by suppressing Src/Stat3 signaling. (**A**) Cell viability assay of A431-III cells treated with Lu, Qu, Su6656 and S3I-301 using an (3-(4,5-Dimethylthiazol-2-yl)-2,5- diphenyltetrazolium bromide (MTT) assay. (**B**) Western blot analysis of the protein levels in A431-III cells after treatment with 10 and 20 μM of Lu and 20 and 40 μM of Qu. (**C**) Same as (**B**), but with 1, 5 and 10 μM of Su6656. (**D**) Same as (**B**), but with 100, 200 and 400 μM of S3I-201.

In our earlier studies, we observed that Lu and Qu significantly inhibited the proliferation of A431 cells with IC_50_ values of 19 and 21 μM, respectively [14]. In a separate study, we found both Lu and Qu dose-dependently (10–100 μM) inhibited MiaPaCa-2 cellular protein kinase activities, and the estimated IC50 of Lu and Qu were 22 and 14 μM, respectively [14]. We concluded that 10 to 40 μM is an adequate concentration for studying the effects of Lu and Qu on cancer cells. Since then, this concentration was used in most of our studies. In our earlier report, the flavonoids, Lu and Qu, inhibited Src/FAK signaling to decrease the migration and invasion of cancer cells [41]. To analyze the regulation signaling of S100A7 in A431-III cells, we analyzed the protein level of Src, p-Src, Stat3, p-Stat3 and S100A7 after treatment with Lu, Qu, Su6656 (a Src inhibitor) and S3I-201 (a Stat3 inhibitor). We analyzed the cell viability of A431-III cells after treatment with Lu (Figure 2Aa), Qu (Figure 2Ab), Su6656 (Figure 2Ac) and S3I-201 (Figure 2Ad). Treatment with 10 and 20 μM Lu and 20 and 40 μM Qu show a decrease in the protein levels of S100A7, phosphorylated Src (p-Src) and p-Stat3 (Figure 2B). After further treatment of A431-III cells with 1, 5 and 10 μM of Su6656, the protein levels of S100A7, p-Src and p-Stat3 were again reduced (Figure 2C). After treatment of A431-III cells with 100, 200, and 400 μM of S3I-201, protein levels of S100A7 and p-Stat3 were also reduced (Figure 2D). These results suggest that Lu and Qu inhibited Src/Stat3 signaling to decrease the expression of S100A7.

### 3.3. Transactivation Activity of S100A7 is Regulated by Src/Stat3 Signaling

**Figure 3:**
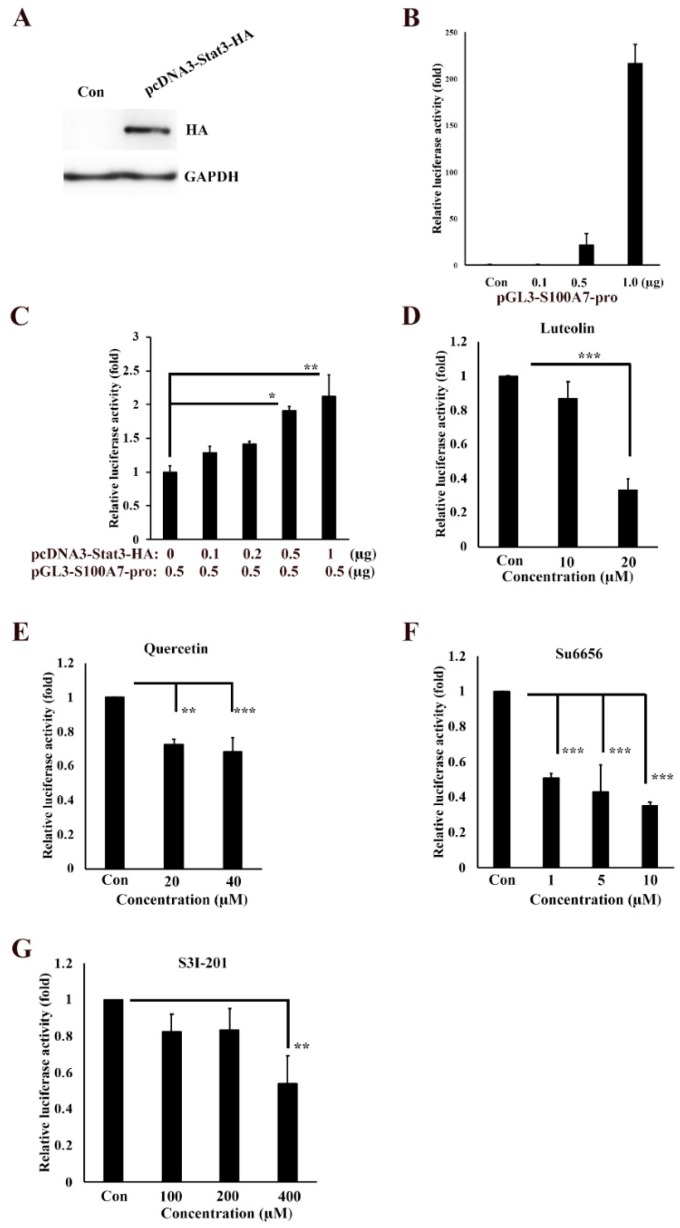
Transactivation activity of S100A7 is regulated by Src/Stat3 signaling. (**A**) Western blot analysis of the protein level of Stat3 after transfection with pcDNA3-Stat3-HA plasmid in A431-III cells. (**B**) Transactivation activity of the 5′-upstream regions of *S100A7*, analyzed by luciferase assay in A431-III cells. Transactivation activity of the 5′-upstream regions of *S100A7* was activated by Stat3 (**C**), but inhibited by luteolin (**D**), quercetin (**E**), Su6656 (**F**), and S3I-201 (**G**). Statistical significance between groups was analyzed by a one-way ANOVA with Tukey’s test (* *p* < 0.05, ** *p* < 0.01, and *** *p* < 0.001).

To further analyze the regulation signaling of S100A7 in cancer cells, we isolated the 5′-upstream regions of S100A7 as promoter and cloned it to the pGL3-Basic vector, creating the pGL3-S100A7-Luc plasmid. The coding regions of *stat3* gene was also isolated and cloned to pcDNA3-HA to create the pcDNA3-stat3-HA plasmid, followed by transfection into A431-III cells and examination of the protein levels of S100A7 by Western blotting (Figure 3A). Transfection of 0.1, 0.5 and 1 μg of pGL3-S100A7-Luc into A431-III cells produced 0.9-, 22- and 217-fold higher transactivation activities of S100A7 promoter, respectively, compared to that with the pGL3-Basic-transfected plasmid (Figure 3B). Transfection of 0.5 μg pGL3-S100A7-Luc plasmid combined with 0.1, 0.2, 0.5 and 1 μg pcDNA3-stat3-HA plasmid in A431-III cells increased the transactivation activity of S100A7 promoter by 1.3-, 1.4-, 1.9- and 2.1-fold, respectively, compared to that with the pGL3-S100A7-Luc plasmid alone (Figure 3C). By further transfection with 1 μg of the pGL3-S100A7-Luc plasmid, treatment with 10 and 20 μM of Lu decrease of transactivation activity of S100A7 promoter to 87% and 33%, respectively, compared to the control group (Figure 3D). Treatment with 20 and 40 μM of Qu decreased the transactivation activity of S100A7 promoter to 73% and 68%, respectively (Figure 3E). Treatment with 1, 5 and 10 μM of Su6656 also decreased the transactivation activities of S100A7 promoter to 51%, 43%, and 35%, respectively (Figure 3F). Treatment with 100, 200 and 400 μM S3I-201 decreased the transactivation activity of S100A7 promoter to 82%, 83%, and 54%, respectively (Figure 3G). These results suggest that Lu and Qu inhibited Src/Stat3 signaling to decrease the transactivation activity of S100A7.

### 3.4. Src/Stat3 Signaling Regulates the Migratory Ability of A431-III, which is Reduced by Lu and Qu

**Figure 4:**
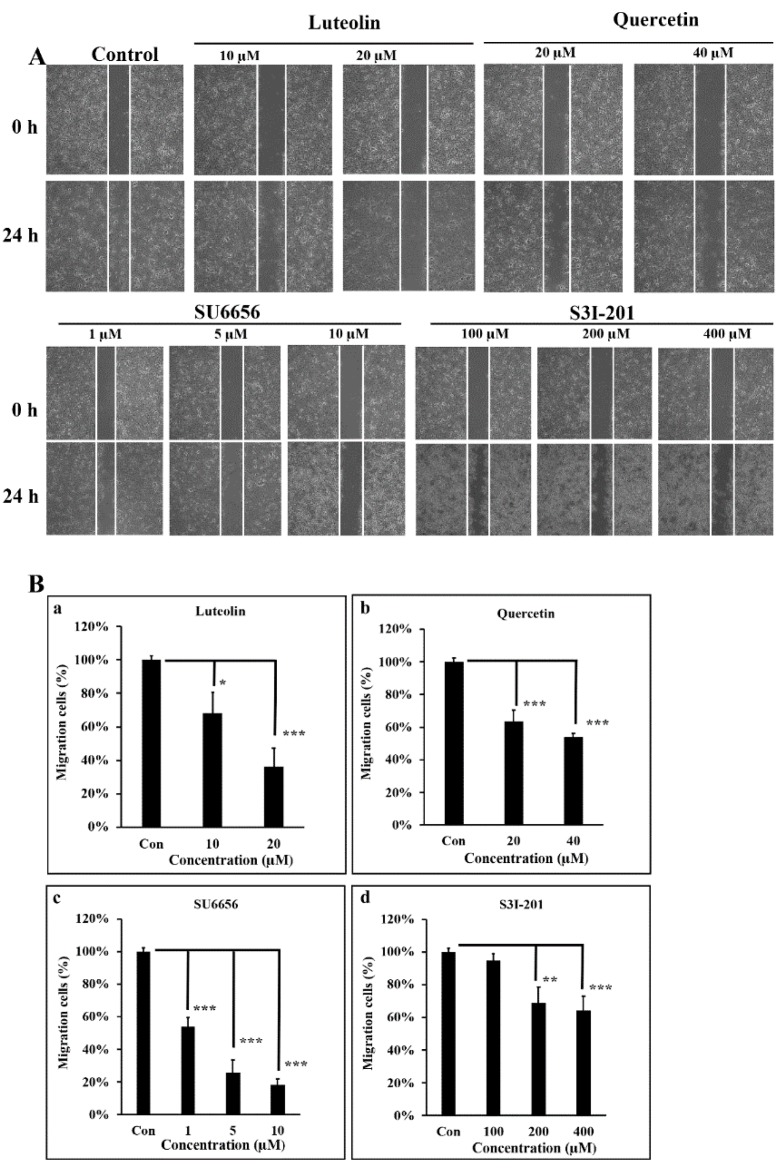
Src/Stat3 signaling regulates the migratory ability of A431-III cells, which was reduced by the effectors. A wound-healing assay was conducted, and the cells were observed to migrate into the wound area. (**A**) A431-III cells were treated with dimethyl sulfoxide (DMSO), 10 and 20 μM of luteolin, 20 and 40 μM of quercetin, 1, 5 and 10 μM of Su6656, and 100, 200 and 400 μM of S3I-201. (**B**) The numbers of migrating cells treated with luteolin (a), quercetin (b), SU6656 (c) and S3I-201 (d) were measured from (A) and analyzed using ImageJ software (NIH, Bethesda, MA, USA). Statistical significance between groups were analyzed by a one-way ANOVA with Tukey’s test (* *p* < 0.05; ** *p* < 0.01; *** *p* < 0.001).

In our earlier reports, we clearly demonstrated that A431-III cells, compared to A431-P cells, show higher migratory capacity and greater invasive potential [2,26,27,28,29,30,41]. To further characterize the migratory ability of A431-III cells as regulated by Src/Stat3/S100A7 signaling, we employed a wound-healing experiment. Treatment A431-III cells with 10 and 20 μM Lu for 24 h decreased the cell migratory abilities to 68% and 36% (Figure 4A,Ba). Treatment of A431-III cells with 20 and 40 μM Qu decreased the migratory ability to 63% and 54%, respectively (Figure 4A,Bb). Treatment of A431-III cells with 1, 5 and 10 μM Su6656 decreased the migration activity to 54%, 26%, and 18%, respectively (Figure 4A,Bc). Treatment with 100, 200 and 400 μM S3I-201 decreased the migratory abilities to 95%, 69%, and 64%, respectively (Figure 4A,Bd). These data suggest that Lu and Qu inhibited Src/Stat3 signaling to decrease the migratory abilities of A431-III cells.

### 3.5. Reduction of Src/Stat3 Signaling Inhibits the Invasive Ability of A431-III Cells

**Figure 5:**
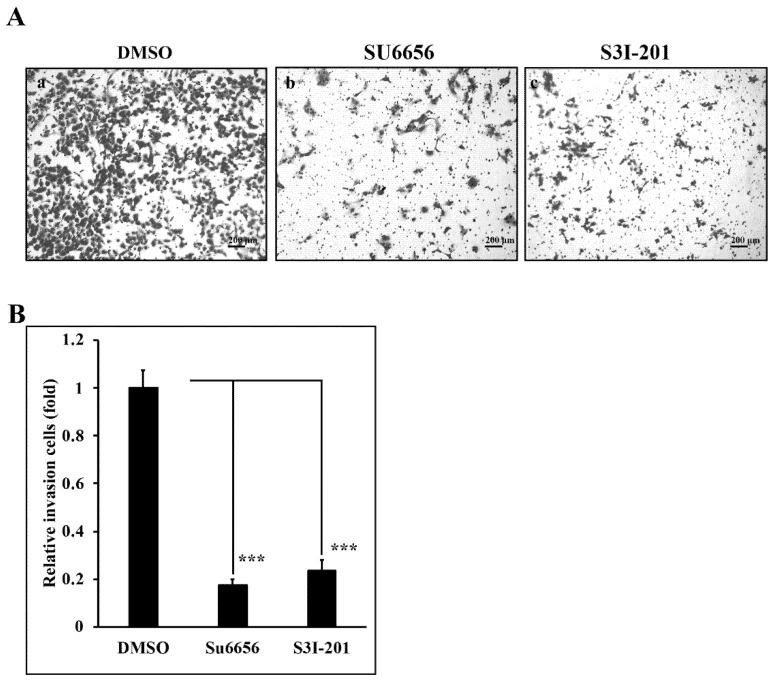
Src/Stat3 regulates the invasive ability of A431-III cells. (**A**) A trans-well assay was used to analyze the invasive ability of A431-III cells after pretreatment with 0.1% of DMSO (**Aa**), 10 μM of Su6656 (**Ab**), and 400 μM of S3I-201 (**Ac**) for 24 h prior to seeding. (**B**) The number of invasive cells were calculated and analyzed by ImageJ software. Statistical significance between groups was analyzed by a one-way ANOVA with Tukey’s test (*** *p* < 0.001).

In our previous reports, we found that Lu and Qu could reduce the invasive ability of A431-III cells [2,27,28,29,30,41]. To analyze the invasive ability regulated by Src/Stat3 signaling, we prepared an invasion assay. A431-III cells treated with 10 μM Su6656 (Figure 5Ab,B) and 400 μM S3I-201 (Figure 5Ac,B) for 24h exhibited a reduction of invasive ability to 18% and 23%, respectively, compared to 0.1% DMSO treatment (Figure 5Aa,B). These results show that Src/Stat3 signaling activates the invasion ability of A431-III cells.

### 3.6. Src/Stat3/S100A7 Signaling Activates the EMT Signaling in A431-III Cells

**Figure 6:**
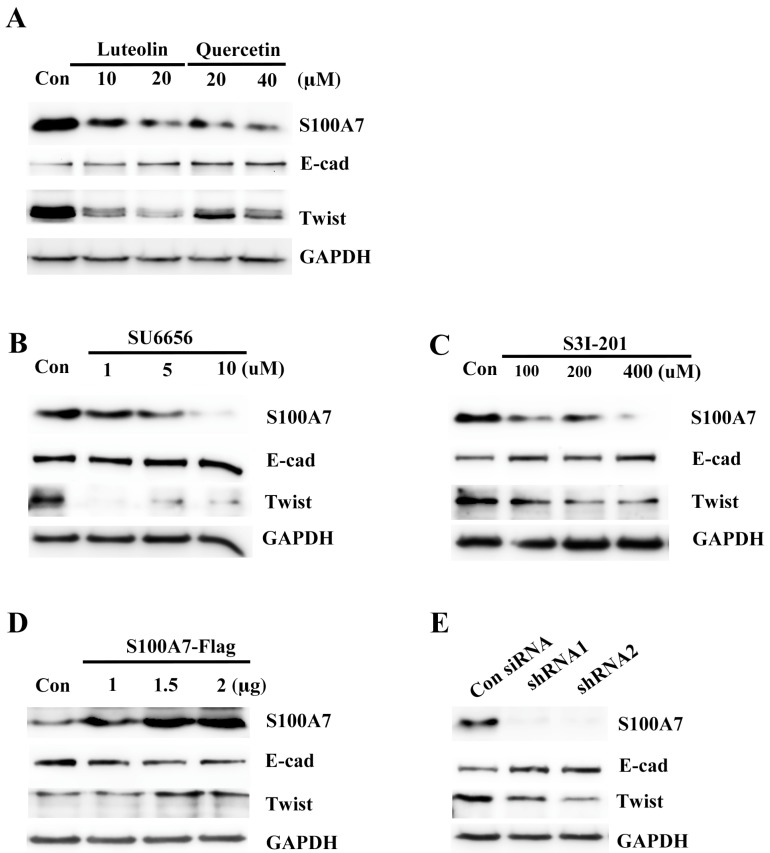
Src/Stat3/S100A7 signaling activates the epithelial-mesenchymal transition in A431-III cells. The protein levels of E-cadherin and Twist were analyzed by Western blot after treatment with 10, 20 μM of luteolin and 20, 40 μM of quercetin (**A**); 1, 5, 10 μM of SU6656 (**B**); or 100, 200, 400 μM of S3I-201 (**C**); and after overexpressing S100A7 in A431-P cells (**D**); or knockdown of S100A7 in A431-III cells by S100A7 shRNAs (**E**).

In earlier reports, A431-III cells were shown to become highly migratory and invasive through EMT signaling [27,28,29,30,41]. S100A7 activated migratory and invasive abilities through the EMT signaling in cancer cells [38,40]. To make clear whether Src/Stat3 signaling activated S100A7 expression and contributed to activation of EMT signaling, we analyzed EMT proteins in A431-III cells after treatment with Lu, Qu, Su6656 and S3I-201. Treatment with 10 and 20 μM of Lu and 20 and 40 μM Qu increased E-cadherin protein and decreased Twist protein (Figure 6A). Treatment with 1, 5 and 10 μM Su6656 increased E-cadherin protein and decreased Twist protein (Figure 6B). Treatment with 100, 200 and 400 μM of S3I-201 increased E-cadherin protein and decreased Twist protein (Figure 6C). Conversely, further transfection with 1, 1.5 and 2 μg of the pcDNA3-S100A7-HA plasmid reduced E-cadherin protein and increased Twist protein (Figure 6D). Knockdown of S100A7 with two shRNAs in A431-III cells increased E-cadherin protein and decreased Twist protein (Figure 6E). These results suggest that Lu and Qu inhibited Src/Stat3/S100A7 signaling to decrease activation of EMT signaling.

### 3.7. Metastasis of A431-III Cells was Reduced by Suppression of Src/Stat3/S100A7 Signaling in Zebrafish

**Figure 7:**
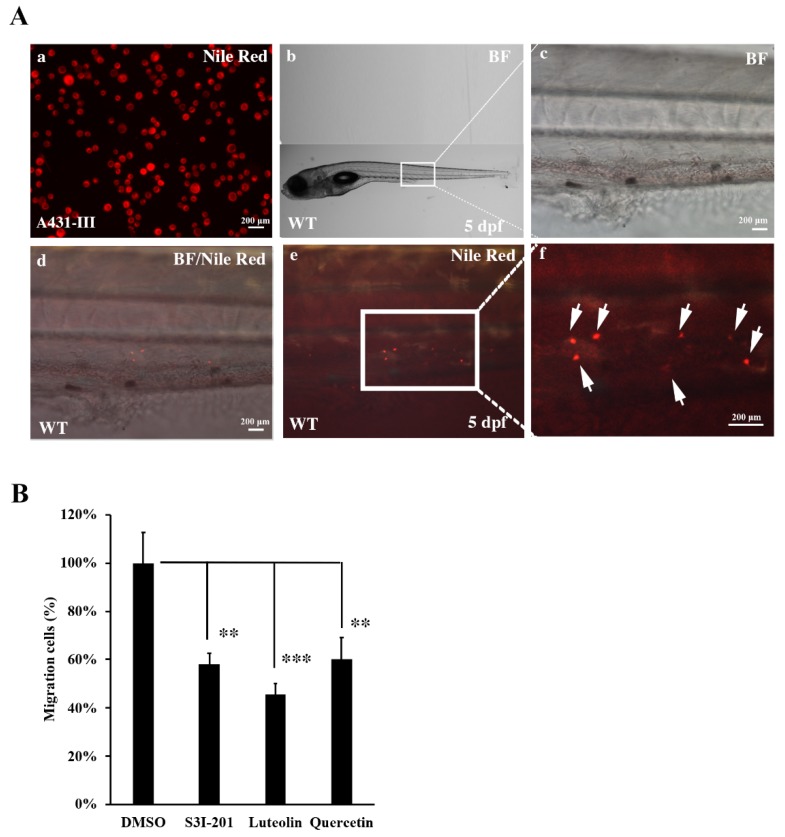
Metastasis of A431-III cells were reduced by suppression of Src/Stat3 signaling in zebrafish. (**A**) A431-III cells were stained with Nile red and microinjected into the pericardiac space of zebrafish larvae at 2 days post-fertilization (dpf). (a) A431-III cells were stained with Nile-Red. (b) The bright field view of 5-dpf zebrafish larvae. Bright field view (c), bright field combined with fluorescent view (d) and fluorescent view (e) of migrative tumor cells enlarged from figure b. (f) Fluorescent view of migrative tumor cells (white arrow) enlarged view from figure e. The number of migrative tumor cells were measured by fluorescent microscope. (**B**) Measurement of metastatic tumor cell numbers pretreated with 0.1% DMSO (DMSO), 400 μM S3I-201 (S3I-201), 20 μM luteolin, and 40 μM quercetin in zebrafish larvae. Statistical significance between groups were analyzed by a one-way ANOVA with Tukey’s test (** *p* < 0.01; *** *p* < 0.001). BF: bright field.

To analyze the role of Src/Stat3/S100A7 signaling in the metastasis of cancer cells in vivo, A431-III cells were microinjected into zebrafish larvae and the metastatic tumor cells were measured. A431-III cells were pre-treated with DMSO, S3I-201, Lu and Qu for 24h, followed by staining with Nile red for microinjection into zebrafish larvae at 2 dpf. The tumor cells migrated to caudal vein beyond yolk extension in 5-dpf zebrafish larvae (Figure 7A, white arrow). In our data, we had found treatment with 20 μM of Lu, 40 μM of Qu and 400 μM of S3I-201 decreased the migration and invasion abilities of cancer cells. To analyze the metastatic ability of tumor cells in the zebrafish model, A431-III cells were pre-treated with 400 μM of S3I-201, 20 μM of Lu and 40 μM of Qu; with those treated with 0.1% DMSO, the numbers of metastatic tumor cells were also reduced to 59, 47 and 61% (Figure 7B). These results show that Lu and Qu can inhibit Src/Stat3/S100A7 signaling to decrease the metastasis of tumor cells in vivo.

## 4. Discussion

S100A7 was shown to activate metastasis of tumor cells and showed a negative correlation with survival rates in cancer patients [40,45]. Overexpression of S100A7 increased tumor progression and metastatic ability in several cancer cell lines. Microenvironmental factors, such as the EMT markers pro-MMP9 and active MMP9, were also activated by S100A7 [40,46]. S100A7 was shown to play an important role during tumorigenesis and metastasis. Until now, no S100A7 inhibitor was found. Understanding the regulation signaling of S100A7 in cancer cells might provide a way of developing new therapeutic strategies.

To investigate the mechanism of metastasis of cancer cells, we used a highly invasive cancer cell model to analyze regulatory signaling. Src/FAK/p-cortactin, Akt/mTOR/c-Myc, UBE2S/hypoxia, and reactive oxygen species (ROS) signaling activated and promoted the metastasis of A431-III tumor cells [2,27,28,29,30,41,43]. Herein, we analyzed the expression and regulation of S100A7 in metastasis using a highly invasive A431-III tumor model. The mRNA and protein levels of S100A7 were higher in A431-III cells than in A431-P cells. A431 cells initiated the expression of S100A7 protein during its in vivo growth in athymic mice [46]. These observations suggest that S100A7 might play an important role in the metastasis of squamous carcinoma cells.

To investigate the regulatory signaling of S100A7 in the metastasis of cancer cells, we analyzed the 5′-upstream regions of *s100a7* gene and found one Stat-binding site. In our earlier study, we found activation of Src signaling in A431-III cells [41]. Stat3 activation by Src leading to malignant transformation was also documented [47]. Therefore, S100A7 might be activated by Src/Stat3 signaling. In the above analysis, A431-III cells show higher levels of p-Src, p-Stat3, and S100A7 than in A431-P cells. We suggested that Src/Stat3 signaling activated S100A7 in A431-III cells.

In earlier reports, luteolin (Lu) and quercetin (Qu) inhibited the metastasis of A431-III cancer cells [27,29,30,41]. Treatment of A431-III cells with Lu and Qu deceased the protein level of S100A7, p-Src and p-Stat3. When using the Src inhibitor SU6656 to treat the A431-III cells, there were reductions in the protein levels of p-Stat3 and S100A7. Furthermore, treatment of A431-III cells with another Stat3 inhibitor S3I-201 decreased the protein level of S100A7. These results suggest that Src/Stat3 signaling activated S100A7. To further analyze the activation signaling of S100A7 by Stat3, we identified and isolated the 5′-upstream regions of the *S100A7* gene. Overexpression of Stat3 in A431-III cells induced transactivation activity of the 5′-upstream regions. Treatment of A431-III cells with SU6656, S3I-201, Lu and Qu decreased the transactivation activity of S100A7 promoter. Taken together, these observations showed that Src/Stat3 signaling activated S100A7 expression.

The role of S100A7 in the metastasis of cervical cancer has been well documented in an earlier report [40]. We further analyzed whether the Src/Stat3/S100A7 signaling was involved in activating the metastatic ability of A431-III cells. Treatment of A431-III cells with SU6656, S3I-201, Lu and Qu decreased the migratory and invasive abilities of A431-III cells according to a wound-healing and invasion assay. Further analysis showed that treatment with Lu, Qu, SU6656 and S3I-201 decreased the protein levels of the EMT markers E-cad and Twist. Overexpression of S100A7 increased the protein level of Twist but reduced the E-cad level, and knockdown of S100A7 had the opposite effects. Based on these results, we suggest that Src/Stat3/S100A7 signaling contributes to the metastasis of cancer cells through the EMT pathway.

Recently, zebrafish have been shown as a metastasis model of cancer cells [48]. The visible zebrafish embryo has the advantage of being able to analyze the metastasis of human tumor cells with fluorescent labeling. The establishment of microinjection technology in zebrafish larvae provided the xenotransplantation of human tumor cells in zebrafish larvae to investigate the intravasation and extravasation of human tumor cells [49,50,51]. Defection of cell immunity in the early stage increased the survival rate of tumor cells [52]. Although the growth temperature was different in zebrafish and human tumor cells, increasing the temperature to 32–34 °C showed both were stable [53,54]. Herein, we used zebrafish to analyze the metastasis of cancer cells in vivo. After treatment of A431-III cells with S3I-201, Lu, Qu or DMSO, the cancer cells were trypsinized, stained with Nile red and then microinjected into 2-dpf zebrafish larvae. Treatment with S3I-201, Lu and Qu decreased the migrative cancer cells at 5-dpf zebrafish larvae.

## 5. Conclusions

This study shows that Src/Stat3 signaling activated S100A7 to enhance the metastatic ability of A431-III cells through the EMT pathway both in vitro and in vivo. Lu and Qu inhibit Src/Stat3/S100Ay signaling to decrease the metastasis of cancer cells.
